# A Practical Treatise on the Diseases Peculiar to Women

**Published:** 1855-08

**Authors:** 


ART. IV.—*A Practical Treatise on the Diseases Peculiar to Women.* Illustrated by cases derived from Hospital and Private Practice. By SAMUEL ASHWELL, M. D., M. R. C. P., etc., etc. Third American from the Third and Revised London edition. Philadelphia: Blanchard and Lea. 1855.

*The Pathology and Treatment of Leucorrhœa.* By W. TYLER SMITH, M. D., M. R. C. P., etc., etc. Philadelphia: Blanchard and Lea. 1855.

It cannot be possible that Messrs. Blanchard and Lea have sent us these handsome volumes in the expectation that we should commend both of them to the favor and pockets of our readers.

We have spent the better part of a hot day on a sofa, comparing their discrepancies. Everything on this vexed subject of uterine disease partakes of a polemic character. There has been a world of false theory, fruitless discussion, disputation, personal abuse, bragging, and second-rate eloquence, expended on the os tincae of late. The uterus is doubtless a very important organ in a variety of senses, but that is no reason why its pear shape should be changed to that of an apple of discord.

To come to our subject. Dr. Ashwell, who "stands by the fathers," is not inclined to admit any new fangled notions of uterine pathology. He has not the eyes of a Bennett to discover ulceration. On page 133 he says: "I have often, in common leucorrhœa, examined by the vagina, but without discovering more than very trifling increase of the body of the uterus, some tenderness of the cervix in the inflammatory form, but none in the protracted or chronic variety. * * * * * * *In no case where there was not suspicion of a venereal taint have I seen erosion or ulceration.*"

*Italics* our own, of course. Leucorrhœa, with Dr. Ashwell, is not an inflammatory disease, it exists from a variety of causes left to the acuteness of the reader to divine; probably by "visitation of God." Leucorrhœa is not vaginal or uterine—it is "acute and mild," or "chronic and aggravated;" a nomenclature, the originality, accuracy and precision of which will commend itself—to the old grannies. "*The secretion from the glands of the interior of the cervix uteri* is not often found in common leucorrhœa," says Dr. Ashwell, indeed that secretion he thinks never exists except in pregnancy. Then have we oftentimes, with upturned nose, looked at the "bird-lime" on our index finger with very mistaken ideas of the source from whence it came.

To sum up Dr. Ashwell's pathology of leucorrhœa. His definition is

"An excessive and altered secretion of the mucus, furnished by the membranes lining the vagina and uterus, by the follicles of the interior of the cervix uteri, and by the lacunæ of the vestibulum; generally white or nearly colorless and transparent; usually without much odor, glutinous, muco-purulent, or purulent; sometimes yellow, green, or slightly sanguineous; and of varying degrees of consistency. The amount of constitutional derangement depending on the severity of the affection and the susceptibility of the patient."

That is comprehensive, almost as much so as the same gentleman's definition of hysteria, viz: "An assemblage of symptoms, generally in a paroxysmal form, simulating many and opposite diseases!" If we understand Dr. Ashwell's notions of leucorrhœa, it is an increased discharge dependent on hyperæmia of the parts—one or all. This is the pathology of the mild form; in the "aggravated," he admits that, matters having gone on from bad to worse, "inflammation" may be said to be present, but never ulceration or abrasion of the os uteri. He takes occasion repeatedly to separate the two ideas of uterine inflammation and leucorrhœa, as entirely disconnected. Speaking of inflammation of the cervix uteri, he says: "As this affection is confined to the glandular part of the uterus, and as it is attended by a peculiar discharge *which rarely forms a part of the common leucorrhœal secretion*, it is entitled to distinct consideration. * * * * * * Out of nearly one thousand cases of sexual disease, treated at Guy's, I find inflammation of the os and cervix has happened only *twenty times*."

Just above we learn *how* he discovered this fact. "From the pain which patients in the ward complain of low down about the sacrum and coccyx, and deeply seated behind the pubis, I have thought there must be inflammation of the cervix; and yet *on examination, although the finger has been covered by a white secretion*, there has been no acute suffering from pressure on the neck of the uterus."

Had Dr. Ashwell looked at the uterus instead of his finger, he might have reached a different conclusion. Had he studied the sensibility of the os uteri, he would not have looked for "acute suffering from pressure."

We turn now to Dr. W. Tyler Smith. On page 79, Dr. Smith, after quoting the views of Dr. Ashwell on the pathology of leucorrhœa, remarks that "Dr. Ashwell is well known as a physician of large experience, and the fullest writer in this country on the subject of leucorrhœa"

But it would be difficult to find any stronger argument for the revision of the whole subject, than that afforded by the uncertainties and errors contained in the extracts just made. Almost every one of the statements they contain requires modification. Nothing relating to leucorrhœa is more common than to find the secretion from the glands of the cervix in ordinary leucorrhœa. *Indeed the secretion from these glands constitutes the most prevalent variety of leucorrhœa.*

Dr. Smith very properly remarks that "many of these erroneous views do not originate with Dr. Ashwell, but have been accumulated from many sources in the absence of anything like exact or positive information."

Dr. Smith may justly lay claim to the credit of furnishing "exact and positive information" relative to the general anatomy and physiology of the tissues involved in leucorrhœa. We are not disposed, however, to accept his pathology. It is, perhaps, too much to expect a London man to coincide fully with Bennett. But we shall speak of this further on, and in the meantime give a hasty *resumé* of the views of Dr. Smith, on the minute anatomy of the vagina and cervix uteri.

The sebaceous follicles and fat glands of the ostium vaginæ are not conconcerned in leucorrhœa to any extent. Their secretion is acid, plentiful during coition, containing butyric acid, and subserving the purpose of lucubration. In this category are to be included the vulvo-vaginal glands of Huguier, which are not infrequently the seat of abscess.

*The Vagina.* The mucous membrane of the vagina, Dr. Smith finds to be covered with a tesselated epithelium, and to partake fully as much of the character of skin, as of mucous tissue. Its villi, or papillæ, are most numerous in the lower part of the canal. They open through the epithelium in the same manner as the sweat glands on the skin. As in the case of all other squamous epithelium, the secretion is acid, an important fact in explaining many of the phenomena of local disease. These villi, with their epithelium, are the parts concerned in vaginal leucorrhœa. The secretion is an exaltation of the natural plasma, accompanied by a rapid desquamation of epithelial scales, constituting what is known as the epithelial discharge. In this form of discharge we shall find, according to the severity of the case,

1. Acid Plasma.
2. Scaly Epithelium.
3. Pus Corpuscles.
4. Blood Globules.
5. Fatty Matter.

Of these the first two are constant, the third and fourth dependent on marked inflammatory action, and the fifth constant, but in varying proportion.

It is enough to say at present, without explanation, that a simple vaginal leucorrhœa should be quite fluid, not at all adhesive or gummy, and white, milky or creamy, owing what color it may have to the presence of epithelium.

Those familiar with leucorrhœa will recognize this as perhaps the least frequent variety of vaginal discharge, indeed we may say that it is rarely found unmixed.

*Cervix Uteri.* The scaly epithelium is continued from the vaginal membrane over the vaginal part of the os; but immediately on merging into the canal of the cervix, it changes its character from the scaly to the columnar, or rather to the ciliated columnar variety. The villi assume the form, but not the sensibility of sensitive papillæ, being larger, more rounded, and less filiform than the villi of the vagina. The marked resemblance of these villi to the tactile papillæ of the skin has misled many observers into supposing that their functions were identical. The microscope, however, has not succeeded in tracing nerves to them, and they are most largely developed where sensation is most deficient.

These reasons, together with the large supply of blood sent to them, are sufficient to warrant Dr. Smith in supposing that their office is purely secretory, that they supply the thick columnar epithelium, together with the firm albuminous secretion found within the os.

This secretion is alkaline. What, now, are the pathological deductions?

Leucorrhœa of the cervix uteri must furnish an exaggeration of the normal secretion. The albuminous, alkaline secretion of the cervix is poured down the vagina in increased quantity, together with the debris of the ciliated epithelium, and on its passage becomes coagulated by the acid vaginal secretion. This, then, is the source of those curdy bodies found in leucorrhœa. We shall have, therefore, as the elements of cervical leucorrhœa,

1. Alkaline Plasma.
2. Mucous Corpuscles.
3. Altered Cylinder Epithelium.
4. Pus Corpuscles.
5. Blood Globules.
6. Fatty Particles.

We regard these observations of Dr. Smith's, as extremely important in settling the question of the frequency of uterine leucorrhœa. We are willing, also, to concede to them a large share of originality — at any rate, we have not met with the same information elsewhere. We cannot pass by the physiological inquiries of Dr. Smith, without noticing his views of the importance of the albuminous plug of the canal of the cervix, in the diagnosis of pregnancy.

Up to the period of pu[illegible]ty this plug remains unmoved, and the secretion from the cervical villi is c[illegible]y sufficient to maintain its integrity from loss by debris. The upper part of this plug is clear and translucent, the lower part white and opaque from coagulation of its albumen by the acid of the vaginal secretion. On every occurrence of menstruation it is washed out by that discharge, and again renewed in the interval. During pregnancy the form of the plug is accommodated to the changes in the size and shape of the os. It becomes shorter and wider, gradually enlarging its horizontal diameter. At the outset of parturition it dissolves and furnishes a part of the lubricating fluid then so abundantly poured out from the cervix uteri, and not — says Dr. Smith — from the vagina.

The application of this is evident, and as Dr. Smith shows by illustrative cases, the presence of the broad white plug at the os uteri, visible under the speculum, might serve as a very important evidence of pregnancy in doubtful cases.

With so much that is valuable in Dr. Smith's monograph, we find ourselves at a loss to account for his idea of the connection of leucorrhœa and inflammation. He makes inflammation the consequence and result of leucorrhœa, a position which we should reverse. But we have no time or space left for its discussion, and so simply enter our protest.

We have made little mention of Dr. Ashwell since taking up Dr. Smith. The two books are antipodes. One is a record of the impressions of a large experience, unrecorded, unanalyzed, unaided by the speculum, or by physiological inquiry. We have but little faith in any man's memory of experience; he must record it to gain our confidence.

The other work — Dr. Smith's — is the result of inquiry, observation, aided by the speculum, the microscope, the test-tube; a record of facts worked up by a clear, accurate, and logical mind. It has a positive value to the practitioner, and though we cannot in all respects endorse its pathology, we commend it to our readers as a valuable addition to their libraries.

For sale by L. Danforth and by Phinney & Co.

